# Treatment with Molgramostim (Recombinant Human Granulocyte-Macrophage Colony Stimulating Factor, Rhugm-Csf, Mielogen) and Lenograstim (Granulocyte-Colony Stimulating Factor) Improves Experimental Colitis in Rats

**DOI:** 10.1155/2019/8298192

**Published:** 2019-10-09

**Authors:** Apostolos E. Papalois, Calypso Barbatis, Dimosthenis Chrysikos, Maria Korontzi, Michail Sideris, Theodoros Pittaras, Eleni Triantafyllidi, Alexandros Nomikos, John K. Triantafillidis

**Affiliations:** ^1^Experimental, Educational and Research Center ELPEN, Athens, Greece; ^2^European University Cyprus, School of Medicine, Nicosia, Cyprus; ^3^Department of Pathology, Red Cross Hospital, Athens, Greece; ^4^Barts and the London School of Medicine and Dentistry, Queen Mary University of London, London, UK; ^5^Hematology Laboratory – Blood Bank, University of Athens School of Medicine, Aretaieion Hospital, Athens, Greece; ^6^Inflammatory Bowel Disease Unit, IASO General Hospital, Athens, Greece

## Abstract

**Background/Aim:**

Treatment with growth factors could be beneficial in both inflammatory bowel disease and experimental colitis. The aim of this study was to investigate the effect of Colony Stimulating Factor (CSF), and Recombinant Human (rHu) Granulocyte Stimulating Factor (GSF) in experimental colitis in rats.

**Methods:**

Experimental colitis was induced in 62 male Wistar rats, divided into 9 groups, using 2,4,6-trinitrobenzensulfonic acid (TNBS). *Group 1:* Ten rats with colitis without treatment (control group). Euthanasia after 15 days. *Group 2:* Ten animals with colitis without treatment (control group). Euthanasia after 30 days. *Group 3:* Six animals with colitis. Immediate treatment with CSF. Euthanasia after 19 days. *Group 4:* Six animals with colitis. Treatment started 7 days after the induction of colitis. Animals were kept for 19 days. *Group 5:* Six animals with colitis. Treatment started 2 weeks after the induction of colitis. *Group 6:* Six animals with colitis, the same as in group 3. Treatment with GSF. *Group 7:* Six animals with colitis, the same as in group 4. Treatment with GSF.

**Group 8:**

Six animals with colitis, the same as in group 5. Treatment with GSF. *Group 9:* Six animals with colitis. Immediate treatment with prednisolone. Euthanasia after 15 days.

**Results:**

CSF and GSF administration significantly improved the histological score (*P* < 0.05) and reduced malondialdehyde contents (*P* < 0.05), compared to control groups in all animals. CSF was superior to GSF and to prednisolone.

**Conclusion:**

Administration of both CSF and GSF could significantly improve the histological score and oxidative stress in experimental colitis in rats.

## 1. Introduction

Inflammatory bowel disease (IBD) is a chronic and relapsing inflammatory disorder of the gastrointestinal with two main entities, Crohn's Disease (CD) and Ulcerative Colitis (UC). The primary therapeutic goals are improvement of quality of life through induction and maintenance of remission, prediction, prevention, and treatment of complications, restoration of nutritional deficits, and alteration of the natural history of the disease [1]. A dysfunctional innate immune system might significantly be involved in the pathogenesis of CD. Therefore, treatments aiming to overcome innate immunity deficiencies could represent valuable therapeutic alternatives [2].

Recombinant human Granulocyte-Macrophage Colony Stimulating Factor [GM-CSF (rHu GM-CSF)] [molgramostim (Mielogen)] is a human protein produced by a strain of Escherichia coli containing a plasmid encoding the human GM-CSF gene [3]. Cells expressing receptors for GM-CSF include neutrophils, monocytes, macrophages and antigen-presenting cells. These receptors are also produced by specialized intestinal epithelial cells including Paneth cells and CD4 T-lymphocyte cells. Molgramostim stimulates the formation of macrophage colonies being essential for the process of hemopoiesis and the function of mature myeloid cells. Moreover, molgramostim increases the number and enhances the functions of white blood cells, increases phagocytosis against microbes and promotes oxidative metabolism, functions related to the innate immunological defense mechanisms of the host.

Lenograstim is the glycosylated recombinant form of human granulocyte colony stimulating factor with actions similar to those of molgramostim. Lenograstim is a valuable adjunct able to minimise the haematological toxicity of myelosuppressive chemotherapy in patients with malignant disease [4].

Of the currently in use animal models, the 2,4,6-Trinitrobenzenesulfonic acid (TNBS) induced colitis, resembles human IBD in its various histological features, including infiltration of colonic mucosa by neutrophils and macrophages and increased production of inflammatory mediators [5–7]. It might be possible that the immunostimulating functions of growth factors could be of benefit in patients with IBD [2].

Finally, malondialdehyde (MDA), is one of the final products of lipid peroxidation in the cells. An increase in free radicals during an inflammatory process as in colitis, causes overproduction of MDA. As a result, MDA level is commonly used as a marker of oxidative stress.

The aim of this study was to investigate the influence of the two growth factors; Granulocyte-Colony Stimulating Factor (G-CSF, *Lenograstim)*, and recombinant human Granulocyte-Macrophage Colony Stimulating Factor, (rHu GM-CSF, *Molgramostim, Mielogen)*], as possible therapeutic agents in experimental colitis in rats.

## 2. Materials and Methods

### 2.1. Experimental Animals

Sixty-two male Wistar rats of body weight greater than 250* *g were used. Animals were kept in the laboratory for at least 10 days before the experiment according to international regulations. The experimental procedures described below were approved by the Animal Care Committee according to the European Union Act and Greek Law 160, A-64, May, 1991 and were designed to minimise pain and discomfort to the animals. All animals were euthanized by barbiturate overdose (150* *mg/kgr pentobarbital sodium). The selection of various time points at which euthanasia was performed, allowed us to investigate the clinical deterioration or remission of colitis across different time intervals.

### 2.2. Induction of Experimental Colitis

Distal colitis was induced by intracolonic installation of 25* *mg of TNBS dissolved in 0.25* *mL of 50% ethanol. The solution was injected into the colon 8* *cm proximal to the anus with a PE-50 cannula. In order to ensure that TNBS ethanol solution was not immediately expelled by the rat, the cannula was left in place for 15* *s prior to its removal.

### 2.3. Groups

Animals were divided into 9 groups. The first two groups consisted of ten rats each (control groups, 20 animals in total). All other groups consisted of 6 rats each (42 animals in total). The number of animals used per group was based on a similar estimate to the ones that other relevant studies reported to have used across the literature. Those numbers were the highest possible for the available budget to allow safe conclusions. In other words, we recruited the highest possible number of mice for the given budget; we also internally allocated a certain number of mice per group and compared this to other studies with similar orientation to ensure adequate power of the study is achieved within the limitations of the cost.

Prednisolone was selected as a positive control group.

The nine groups were as follows Table 1: *Group 1:* Ten rats with TNBS colitis without treatment (control group 1). Euthanasia was performed after 15 days. *Group 2:* Ten animals with TNBS colitis again without treatment (control group 2). Euthanasia was performed after 30 days. *Group 3:* Six animals with TNBS colitis. Immediate treatment with CSF. Euthanasia was performed after 19 days. *Group 4:* Six animals with TNBS colitis. Treatment with CSF started 7 days after induction of colitis. Euthanasia was performed after 19 days. *Group 5:* Six animals with TNBS colitis. Treatment with CSF started 14 days after the induction of colitis. Euthanasia was performed after 19 days. *Group 6:* Six animals with TNBS colitis, immediate treatment with GSF. Euthanasia was performed after 19 days.* Group 7:* Six animals with TNBS colitis, treatment with GSF started 7 days after induction of colitis. Euthanasia was performed after 19 days.* Group 8.* Six animals with TNBS colitis, treatment with GSF started 14 days after the induction of colitis. Euthanasia was performed after 19 days*. Group 9:* Six animals with TNBS colitis. Immediate treatment with prednisolone and euthanasia after 15 days.

Growth factors were administered every 2 days at a dose of 10 mcg/kg. The dose of prednisolone was 5.3* *×* *10^−3^* *mmol/kg.

### 2.4. Pathology

The resected tissue samples were fixed in 10% buffered formaldehyde, and each specimen was entirely embedded in 3–4 paraffin blocks according to the size. Histological sections from each block were cut in 4* μ*m thickness and stained with Hematoxylin-Eosin [8]. Initially and separately, the sections were blindly examined by two pathologists (CB and AN) and a final agreed histological diagnosis was made. The results were analyzed and evaluated using the Geboes [9] histological score based on the following features: Normal Histology, Acute Ulcers/Erosions/Chronic Ulcers, Neutrophilic Activity, Crypt Distortion and Chronic Inflammation of the lamina propria. The above features, were diagnostic of induced colitis. Based on this score, the large bowel mucosa was characterized as normal (degree 0), colitis in remission (degrees 1 and 2) and active colitis (degrees 3–5). As signs of remission were evaluated the histological features of healing ulcers with complete or incomplete reepithelialization, crypt loss and fibrosis of lamina propria/submucosa, or crypt regeneration.

### 2.5. Tissue Malondialdehyde Estimation

The malondialdehyde****(MDA) measurement was based on the reaction of a chromogenic reagent, *N*-methyl-2-phenylindole (MPI), with MDA at 45°C. The reagents used included Reagent MPI, 10.3* *mmol/L *N*-methyl-2- phenylindole in acetonitrile, MDA standard, 10* *mmol/L 1,1,3,3-tetramethoxypropane in 20* *mmol/L Tris–HCl, 500* *mmol/L butylated-hydroxytoluene, in acetonitrile, 20* *mmol/L Tris buffer pH 7.4, 0.9% NaCl, 37% (12* *mol/L) HCl, methanol, HPLC grade, acetonitrile and HPLC grade. Before the procedure, three volumes of the MPI reagent were diluted with one volume of 100% methanol. Tissue samples were rinsed with ice-cold isotonic saline before homogenization which was carried-out using Tris buffer 20* *mmol/L pH 7.4 and an ULTRA-TURRAX (IKA-Labortechnik) blender. One millilitre buffer was used for 0.1* *g of tissue. Ten millilitres of 500* *mmol/LBHT was added to 1* *mL of tissue homogenate to prevent sample oxidation. The homogenate was centrifuged at 3000 r/min at 4°C for 10* *min. Then 0.2* *mL of sample (plasma or supernatant of tissue homogenate) and 0.65* *mL of diluted MPI reagent were added to a polypropylene microcentrifuge tube. The mixture was vortexed and then 0.15* *mL of 12* *mol/L HCl was added. Tubes were incubated at 45°C for 60* *min and centrifuged at 6000 r/min for 15* *min. Then 0.8* *mL of the supernatant was measured at 586* *nm. MDA standards for the standard curve were made by dilutions of the stock 10* *mm TMOP solution. The final concentrations were 2.08, 4.16, 8.33, 12.5, and 16.66* μ*mol/L and the assay procedure was followed as for the samples. The absorbance was 0.059, 0.124, 0.264, 0.4, and 0.545 respectively [10].

### 2.6. Statistical Analysis

The descriptive data were presented as mean ± standard deviation. Statistical comparisons between groups were made by Pearson's chi square test or Student's *t*-test. Differences were considered statistically significant at the level of *P* < 0.05.

## 3. Results

### 3.1. Pathology

All animals survived during the period of experiment.

*CSF:* the administration of CSF significantly improved the histological score, (colitis in remission) in all groups (3, 4, 5) compared to controls with values *P* = 0.075, *P* = 0.035, *P* = 0.035, respectively (*P* < 0.05) (Table 2). However, improvement in group 5 was less prominent compared with groups 3 and 4, underlying the importance of the early administration of the growth factor.

Differences between CSF and prednizolone were statistically significant in favor of CSF (*P* < 0.05) (Table 2).

*GM-CSF:* the administration of GM-CSF significantly improved the histological score (colitis in remission) (group 6—early administration) (*P* < 0.05). However, improvement was less prominent in the group of delayed administration (group 8). Although GM-CSF improved the histology of the bowel even after one week of induction of colitis, results did not reach the effectiveness of CSF (Table 2).

*CSF vs GM-CSF:* when comparing the two growth factors, CSF was superior to GM-CSF especially in the groups with delayed administration (group 5 vs group 8) *P* = 0.035, *P* = 0.042, respectively (*P* < 0.05).

**Figure 1:**
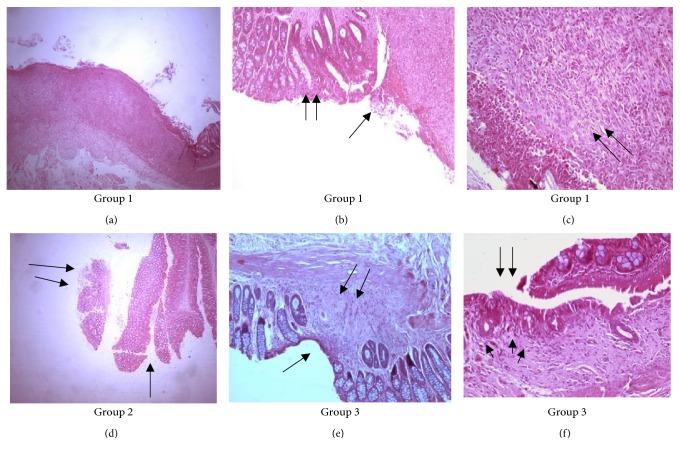
Histological features in Groups 1, 2, 3 depicting lesions of ulcers with surrounded granulation tissue, normal mucosa or healing sites (colitis in remission). (a) extensive chronic ulcer (H/E ×25). (b) Ulcer (arrow) and regenerating adjacent epithelium (arrows) (H/E ×100). (c) Base of ulcer covered by granulation tissue (arrows) (H/E ×200). (d) Normal mucosa (arrow) and acute ulcer (arrows). (e) Healing site of previous ulcer (H/E ×200). Reepitheliastion (arrow) loss of crypts and fibrosis (arrows). (f) Healed site and regenerating crypts (arrow heads) reepithelialization (arrows) (H/E ×100).

**Figure 2:**
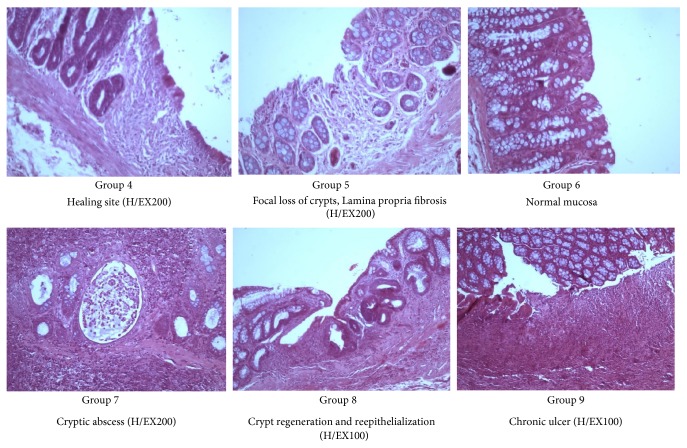
Histological features in Groups 4, 5, 6, 7, 8, 9 depicting healing sites (colitis in remission), cryptic abscess (acute colitis), or chronic ulcers (chronic colitis).

Some representative histological pictures of the various animal groups are shown in Figures 1 and 2.

### 3.2. Tissue Malondialdehyde Levels

The levels of tissue MDA in the treated and untreated animals are shown in Table 3. A statistical significant reduction (*P* < 0.05) in groups (3, 4, 5, 6, 9) versus group 1 (control group),was remarkable. (*P* = 0.017, *P* = 0.011, *P* = 0.030, *P* = 0.044, *P* = 0.023, respectively).

A significant reduction of tissue MDA in treated animals in groups 7 and 8 versus group 1 (control group), was noticed (*P* = 0.272, *P* = 0.316, respectively).

## 4. Discussion

The results of the present study showed that the two growth factors namely Granulocyte-Colony Stimulating Factor (G-CSF, *Lenograstim*) and the recombinant human Granulocyte-Macrophage Colony Stimulating Factor (rHu GM-CSF, *Molgramostim,* Mielogen), exhibited favourable influence on experimental chemical colitis in rats. Both factors improved significantly the histological score compared to control groups. CSF (*Lenograstim*, Granulocyte), produced better results compared to prednisolone and also produces faster healing of the histological lesions compared to *Molgramostim*. Finally, both growth factors exert a significant influence on the oxidative stress accompanying this model of colitis.

Today, treatment of CD with granulocyte colony-stimulating factors should be considered as experimental treatments based on both experimental and clinical studies[11].

Experimental studies dated back to 1996 suggested that prolonged high-dose therapy with G-CSF may have anti-inflammatory effects in colitis [12]. A subsequent study showed that administration of G-CSF at a dose of 250* *µg/kg/day remarkably attenuated both the loss of body weight and colonic wall thickening due to progressive transmural inflammation [13]. Yoshimitsu et al. showed that administration of rhG-CSF prevented the onset of Th1–type TNBS colitis without deterioration of neutrophil-dominant chronic colitis in hosts with higher expression of endogenous G-CSF [14]. Kudo et al. showed that the prevention of epithelial cell apoptosis seems to precede the anti-inflammatory action of rhG-CSF [15].

Human studies have also described beneficial effect of various growth factors including growth hormone, keratinocyte growth factor (KGF), epidermal growth factor (EGF), teduglutide, and GM-CSF/G-CSF in patients with CD. Dejaco et al. found that G-CSF might be efficacious in severe endoscopic postoperative recurrence of CD. Another study showed that patients who were treated with 300* *µg of recombinant human GS-CSF three times per week for 12 weeks showed significant increase in neutrophil counts, IL-1ra, and soluble TNFR p55 and p75 [16].

Korzenik et al. conducted a 12-week open-label trial with filgrastim in 20 patients with moderate to severe CD. Primary end-point was a decrease in the CDAI of >70 points and remission was considered to be a CDAI <150 points. All patients received filgrastim daily for 12 weeks at an initial dose of 300* μ*g sc. The dose was adjusted downward by 100* μ*g if ANC exceeded this range, and after a subsequent reduction to 100* μ*g/day, the dose was lowered to 75* μ*g/day. Five patients (25%) achieved remission during the study, 11 (55%) demonstrated a decrease of at least 70 points, and 3 of 4 (75%) patients with fistulae had a positive response (defined as closure of more than 50% of fistulae). Among responders at week 12, 4/11 (36%) patients maintained response for additional 4 weeks after completion of therapy and the others had an increase in disease activity [17].

GM-CSF has also been tested in patients with active CD. In an open-label dose-escalation trial (4–8 microg/kg/d) 15 patients with moderate to severe CD exhibited a significant decrease in the mean CDAI score by 190 points after 8 weeks of treatment. Overall, 12 patients had a decrease in CDAI of more than 100 points, and 8 achieved clinical remission [18].

Subsequently, Korzenik et al. investigated the role of sargramostim in 124 patients with CD. Patients were randomly assigned to receive sargramostim (6 *μ*g/kgBW) or placebo sc daily for 56 days. The primary end-point (clinical response) was achieved in 54% in the sargramostim group and 44% in the placebo group (*P* = 0.28). However, clinical response and remission rate were significantly higher in the sargramostim than in the placebo group (48% vs 26%, *P* = 0.01 and 40% vs 19%, *P* = 0.01 respectively). The median post-treatment Crohn's Disease Endoscopic Index of Severity score was significantly lower in the sargramostim group [19].

In another clinical trial, patients with corticosteroid-dependent Crohn's Disease were randomised to receive either sargramostim 6 microg/kg sc once daily or placebo for up to 22 weeks. Significantly more sargramostim-treated patients than placebo achieved corticosteroid-free remission as well as corticosteroid-free response[20]. In another study sargramostim at a dose of 6 microg/kg/d improved median CDAI scores, while a minority of patients experienced clinical remission or clinical response [21].

Magno et al. evaluated the safety and efficacy of sargramostin in patients with fistulizing CD who had not responded to conventional therapy or had developed adverse reaction to infliximab. They found that rHu GM-CSF in a dose of 6 microg/kg/d sc for 8 weeks failed to improve the patients. However, the small number of patients and the severity of their disease do not allow drawing firm conclusions about the drug effectiveness [22].

However, a Cochrane meta-analysis of the three available studies with 537 patients showed that sargramostim does not appear to be more effective than placebo for induction of clinical remission or clinical improvement in patients with active CD [23].

Concerning other growth factors, it was suggested that keratinocyte-like growth factor-2 and epidermal growth factor enemas in combination with oral mesalamine, human growth hormone, and sargramostim may have significant synergistic effect in IBD patients [24]. A study aiming to investigate the effect of keratinocyte growth factor gene therapy in acetic acid-induced colitis in rats showed significant improvement in the histological changes [25].

As far as the possible mode of action of these agents is concerned, it has been proposed that growth factors could achieve both healing of the mucosa and restoration of the integrity of epithelium as well [6, 7]. Clinical and experimental observations also suggest that growth factors could restore T-cell and dendritic cell function [11]. Polymorphonuclear apoptosis may be delayed under the influence of G-CSF in the microenvironment of IBD-affected mucosa [26]. G-CSF increases neutrophil tissue migration, which may partially account for its therapeutic effect [27]. Although macrophages are considered a critical factor in determining the severity of acute inflammatory responses in the gut, recent evidence has indicated that macrophages may also play a counter-inflammatory role. M-CSF-dependent macrophages may play either a pro- or counter-inflammatory role in acute experimental colitis, depending on the stimulus used to induce colitis [28]. GM-CSF is expressed by CD4 T cells and Paneth cells in the intestinal epithelium and myeloid cells. Intestinal epithelium cells express GM-CSF receptors throughout the gastrointestinal tract [29]. GM-CSF may overcome defects in neutrophil and macrophage function related to CD. GM-CSF-treated mice showed decreased expression of TNF-a, IL 1-b, IFN-*γ*, and IL1-a [30] and increased production of IL-4, IL-10, and IL-13 [31].

It is well established that the presence of neutrophils among epithelial cells is one of the major features of the inflammation in IBD patients, and has been used to indicate disease activity. The survival of neutrophils outside the blood vessels is limited and their longevity is influenced by GM-CSF, which probably reduces neutrophil apoptosis. Moreover, Griseri et al. emphasized the role of eosinophils as important effectors of IL-23 GM-CSF axis in colitis, the GM-CSF being a strong activator of eosinophilic functions [32].

Overall, our current study aims to shed light on the mechanisms of reduction of oxidative stress in all groups of treated animals, irrespectively of treatment (CSF, GSF, prednizolone). This was achieved by demonstrating reduced levels of tissue malondialdehyde. Although unknown, it could be related to avoidance of oxidant-induced mitochondrial injury [33] or mitochondrial swelling and depolarization [34].

This study has also some limitations. First, the number of the mice in each group was relatively small. Furthermore, the cost of purchasing mice is high and most previous studies have also used relatively small and similar groups.

Finally, another limitation of our study, might be the time of observation which is not related to the clinical outcome.

## 5. Conclusions

In conclusion, the administration of G-CSF and GM-CSF can significantly improve the histological score in experimentally induced colitis in rats. Both factors must be promptly administered in order to achieve maximum therapeutic response. Despite their high economic cost we suggest that these factors could further be tested in patients with acute exacerbation of IBD.

In future clinical trials must be administered as early as possible after the establishment of the acute flare-up of CD.

## Figures and Tables

**Table 1:** Experimental TNBS colitis in 62 male Wistar rats treated with CSF, GM-CSF and prednizolone, divided into 9 groups according to our experimental protocol.

Group	Number of animals with TNBS colitis	Day of euthanasia	Treatment with CSF (started)	Treatment with rHu GSF (started)	Treatment with prednisolone (started)
1	10 (control group, no treatment)	15^th^			
2	10 (control group, no treatment)	30^th^			
3	6	19^th^	Immediate treatment		
4	6	19^th^	7 days after induction of colitis		
5	6	19^th^	14 days after induction of colitis		
6	6	19^th^		Immediate treatment	
7	6	19^th^		7 days after induction of colitis	
8	6			14 days after induction of colitis	
9	6	15^th^			Immediate treatment

**Table 2:** Pathology results divided in three categories, 0: normal mucosa, 1: colitis in remission, 2: active colitis, according to Geboes histological score.

Groups	Category 0 (normal mucosa)	Category 1 (colitis in remission)	Category 2 (active colitis)	Pearson's *Χ^2^* test
1 (Colitis, 15 days, control group)	20% (2/10)	30% (3/10)	50% (5/10)	
2 (Colitis, 30 days, control group)	50% (5/10)	30% (3/10)	20% (2/10)	
3 (Granulocyte)	67% (4/6)	33% (2/6)	0% (0/6)	*P* = 0.075 (vs group 1) vs group 2 *P* = 0.49
4 (Granulocyte)	83% (5/6)	17% (1/6)	0% (0/6)	*P* = 0.035 (vs group 1) vs group 2 *P* = 0.34
				
5 (Granulocyte)	83% (5/6)	17% (1/6)	0% (0/6)	*P* = 0.035 (vs group 1) vs group 2 *P* = 0.34
6 (Mielogen)	50% (3/6)	17% (1/6)	33% (2/6)	*P* = 0.45 (vs group 1) vs group 2 *P* = 0.76
7 (Mielogen)	0% (0/6)	50% (3/6)	50% (3/6)	NS *P* = 0.45 vs group 2 *P* = 0.11
8 (Mielogen)	17% (1/6)	0% (0/6)	83% (5/6)	NS *P* = 0.29 vs group 2 *P* = 0.042
9 (Prednizolone)	33% (2/6)	50% (3/6)	17% (1/6)	*P* = 0.41 (vs group 1) vs group 2 *P* = 0.72

**Table 3:** Mean value of tissue malondialdehyde in treated and untreated groups of animals and 95% confidence interval (mean ± SD, CI) (Comparisons: treated animals versus group 1).

Group	Tissue malondialdehyde (*μ*M/L)	*P*value
1	2.74* *±* *0.36, (2.51–2.96)	
2	2.03* *±* *0.29, (1.85–2.209)	
3	1.12* *±* *0.22, (0.944–1.296)	0.017
4	1.62* *±* *0.31, (1.37–1.868)	0.011
5	1.68* *±* *0.19, (1.52–1.832)	0.030
6	1.71* *±* *0.36, (1.42–1.99)	0.044
7	2.11* *±* *0.43, (1.76–2.46)	0.272
8	1.98* *±* *0.27, (1.76–2.19)	0.316
9	1.72* *±* *0.16, (1.59–1.84)	0.023

## Data Availability

The data used to support the findings of this study are included within the article.
